# A giant pregnancy-associated intra-abdominal desmoid tumour: not necessarily a contraindication for subsequent pregnancy

**DOI:** 10.1186/1477-7819-11-277

**Published:** 2013-10-16

**Authors:** Eelco de Bree, Eustathios Dimitriadis, Elpida Giannikaki, Evangelia G Chryssou, John Melissas

**Affiliations:** 1 Department of Surgical Oncology, Medical School of Crete University Hospital, Heraklion, Greece; 2 Department of Pathology, Medical School of Crete University Hospital, Heraklion, Greece; 3 Department of Radiology, Medical School of Crete University Hospital, Heraklion, Greece

**Keywords:** Aggressive fibromatosis, Desmoid tumour, Intra-abdominal tumour, Pregnancy

## Abstract

Desmoid tumours are rare mesenchymal tumours, often locally invasive and characteristically associated with a high local recurrence rate after resection. A potential aetiological role for female hormones is indicated. Pregnancy-associated desmoid tumours are almost exclusively located in the abdominal wall. An essential issue is how to counsel women who have had a pregnancy-associated desmoid tumour and subsequently wish to bear a child. A considerably rare case of a patient with a resection of a giant pregnancy-associated, 33 cm in diameter, intra-abdominal desmoid tumour is presented. After a subsequent pregnancy, the patient delivered healthy twins 26 months later. Fifty-four months after treatment, there are no signs of recurrent or second desmoid tumour. Although rarely located in the abdomen, pregnancy-associated desmoid tumours should be included in the differential diagnosis of intra-abdominal tumours detected during or shortly after pregnancy. Based on this case and a few others reported in the literature, subsequent pregnancy does not necessarily seem to be a risk factor for recurrent or new disease.

## Background

Desmoid tumour, also called aggressive or desmoid-type fibromatosis, is a rare monoclonal, fibroblastic proliferation. Although histologically benign and unable to give rise to metastases, desmoids are often locally invasive and characteristically associated with a high local recurrence rate after resection. While this fibroblastic disorder may be observed in nearly every part of the body, desmoids occur most commonly in extremities. Intra-abdominal location is observed only in a small proportion of patients [1].

Aetiology of desmoid tumours is incompletely defined. Numerous acknowledged factors are associated with their development [1]. Increased incidence occurring during and after pregnancy, as well as following use of oral contraceptives, the preponderance of women of reproductive age in many series, anecdotal reports of spontaneous tumour regression during menopause, expression of oestrogen beta receptor and reports of tumour regression with anti-oestrogen treatment are all factors which indicate a potential aetiological role for female sex hormones [1,2]. Pregnancy-associated desmoids are almost exclusively located in the abdominal wall [2,3]. Herein we report on a young woman with a giant intra-abdominal desmoid tumour diagnosed 3 weeks after giving birth and resected soon afterwards. The location of a pregnancy-associated desmoid tumour in the abdomen is unusual. Further, due to hormonal influences, a subsequent pregnancy may theoretically be a risk factor for a second desmoid or recurrent disease. Neither during nor after the subsequent pregnancy was recurrence or development of a second desmoid tumour observed in our case. The literature on this topic is herein reviewed.

## Case presentation

**Figure 1:**
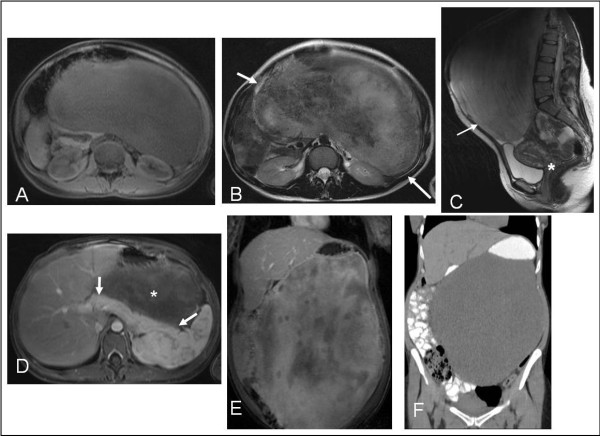
**Radiological images. (A)** Unenhanced T1-weighted MRI shows large, almost homogeneous, well-defined, intermediately low signal intensity mass, occupying the abdominal cavity, displacing solid organs and bowel loops. **(B, C)** T2-weighted MRI depicts heterogeneously hyperintense mass (arrows), with prominent areas of low signal intensity and unrelated to the bladder and uterus (asterisk). **(D, E)** Gadolinium-enhanced T1-weighted MRI shows no intervening fat planes between homogeneously enhancing pancreas (arrows) and slowly and heterogeneously enhancing tumour (asterisk), with intervening low signal intensity areas. **(F)** Unenhanced CT demonstrates the mass displacing opacified bowel loops.

A 31-year-old woman presented with persistent abdominal distension 3 weeks after vaginal delivery of a healthy daughter. It had been her first pregnancy. She was breast-feeding her baby. Her medical history was unremarkable and she had had neither trauma nor any abdominal operation in the past. At physical examination of this generally slim young woman, an enormous mobile intra-abdominal mass was found. Laboratory tests were normal. Computed tomography (CT) and magnetic resonance imaging (MRI) demonstrated a huge solid intra-abdominal soft tissue mass, which occupied most of the peritoneal cavity and which had displaced anatomical structures such as the liver, the pancreas and the small and large intestine (Figure 1). Chest CT showed no evidence of metastatic disease. CT-guided core needle biopsy revealed a mesenchymal, probably myofibroblastic, lesion whose biological behaviour could not be determined.

**Figure 2:**
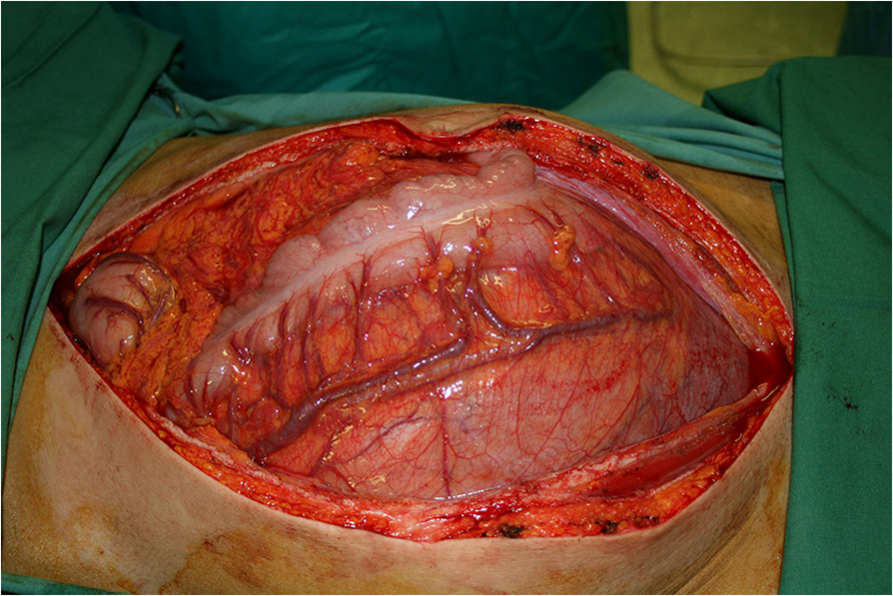
**Findings at laparotomy.** An enormous intra-abdominal tumour was seen adjacent to the overlying transverse colon and its mesocolon.

**Figure 3:**
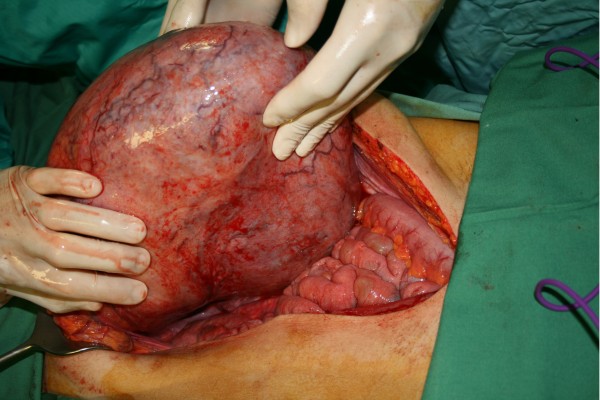
**Intraoperative findings.** Caudally the tumour was mobile and did not involve small bowel loops or pelvic organs.

**Figure 4:**
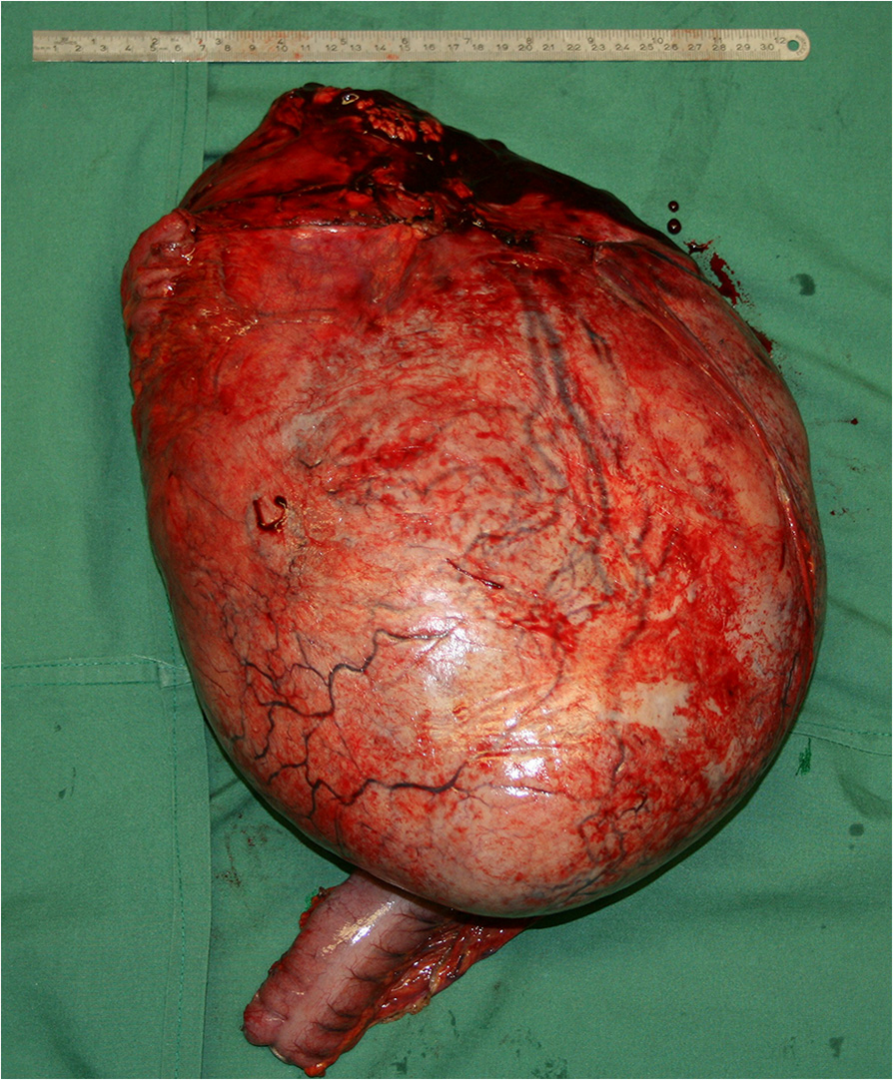
**The surgical specimen.** Dorsal view of the specimen of the giant tumour shows the resected rim of pancreatic tail at the cranial base of the tumour (at the top of the figure). The resected transverse colon overlying the tumour at the frontal site is partially visible.

**Figure 5:**
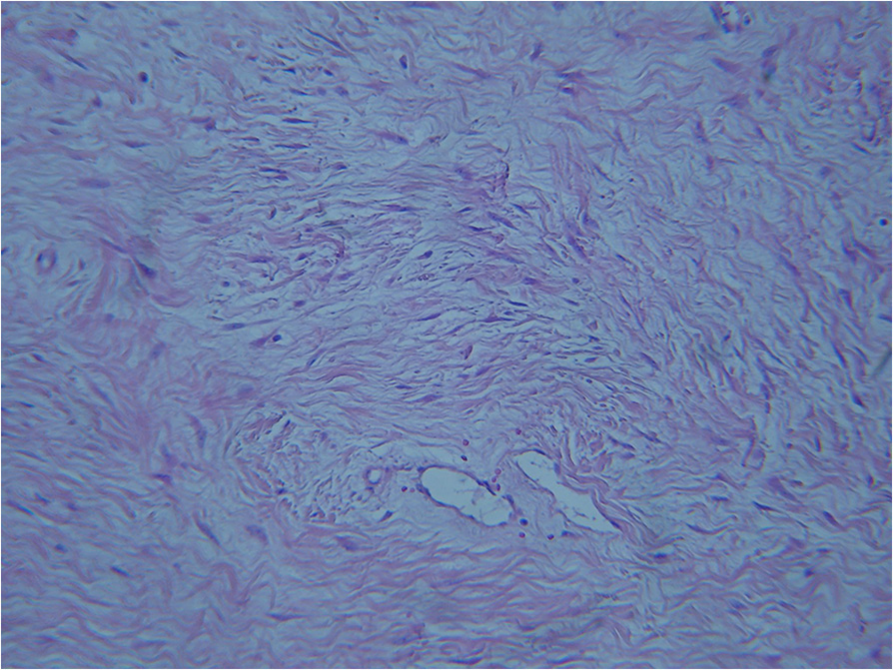
**Histological findings.** Microscopically the tumour was composed of bland, spindle or stellate mesenchymal cells, without obvious atypia or increased mitotic index. The cells were arranged haphazardly in a dense fibrous stroma with thick walled vessels (H&E, ×100).

At laparotomy, a large intra-abdominal mass was observed (Figure 2). The transversal colon and its mesocolon were stretched over the frontal side of the tumour and fixed to the mass (Figure 2). While its caudal side was mobile (Figure 3), the cranial side of the tumour was fixed focally to the inferior part of the tail of the pancreas (Figure 4). There was no infiltration of other organs. The tumour with the transverse colon, its mesocolon and a wide rim of the tail of the pancreas was resected *en bloc*. An end-to-end anastomosis of the ascending with the descending colon was performed. The postoperative course was uneventful. The tumour weighed 6.2 kg and was 33×29×8.5 cm in size. Histological examination demonstrated a mesenteric desmoid tumour (Figure 5) which had actually infiltrated the transverse colon as well as the surface of the resected part of the pancreas. The tumour appeared to have been excised completely. Immunohistochemical staining for oestrogen receptor alpha and progesterone was negative. No pathology was seen in the resected colon.

The patient did not receive any adjuvant treatment. She became spontaneously pregnant, despite the recommendation that she postpone pregnancy to allow for a 2-year disease-free follow-up period. Nevertheless, she delivered healthy twins by Caesarean section 26 months after initial treatment for the desmoid tumour. Fifty-four months after resection of the desmoid, she is in excellent condition without any sign of tumour recurrence on abdominal sonography and MRI, or of development of a second desmoid tumour.

## Discussion

The enormous tumour size in the present case is noteworthy. Primary desmoids >30 cm in size have only incidentally been reported [4-6]. It remains remarkable that the tumour was not observed at routine repetitive sonography during pregnancy. The optimal treatment of desmoids remains difficult to be determined, due to the rarity, the heterogeneity and the very unpredictable natural history of the disease. Therefore, an individualized approach is warranted [1]. Surgical resection has most commonly been used, while radiotherapy, medical treatment and watchful waiting are alternative options [1].

Typically, desmoids arising during or shortly after pregnancy are almost always located in the abdominal wall [2,3]. Such an association with pregnancy has very rarely been observed in other locations (Table 1). Single cases of desmoid tumour arising in the vulva [7], larynx [8], neck [9] and popliteal space [10] during pregnancy have been described, while only a few cases of pregnancy-associated intra-abdominal desmoids, originating from the retroperitoneum [11], mesentery [12,13] and pelvis [14,15] have been reported. In one report [14], a pelvic desmoid tumour interfered both with normal maturation and delivery of the fetus as well as the patient’s ability to void and defecate, prompting surgical intervention at 23 weeks of gestation. Following the resection the patient delivered a healthy full-term baby. In the other pelvic desmoid case [15], the tumour obstructed labour, necessitating a Caesarean section and subsequent tumour excision. In our patient, the intra-abdominal desmoid tumour evidently grew rapidly, reaching its enormous dimensions due to hormonal stimulation during pregnancy. The giant tumour had no apparent adverse influence on the fetus and its vaginal delivery, possibly because the desmoid originated from the transversal mesocolon. Although only a few cases have been reported, desmoid tumour should be included in the differential diagnosis of intra-abdominal tumours detected during or shortly after pregnancy.

**Table 1:** Reports of pregnancy-associated desmoid tumours located outside the abdominal wall

**First author**	**Year**	**Patient’s age (years)**	**Site of origin**	**Size (cm)**	**Time of diagnosis**	**Treatment and outcome**
Ober [10]	1955	18	Popliteal space	3	17 weeks of gestation	Excision on the 12th postpartum day; free of recurrence 1 year later
Allen [7]	1997	19	Vulva	3	5-6 weeks of gestation	Incomplete excision; excision of recurrence after 2 months; second recurrence after several months, treated by excision and radiotherapy
Gherman [8]	1999	25	Larynx	2.3	20 weeks of gestation	Incomplete excision; recurrence after 8 weeks; spontaneous complete regression 9 weeks after delivery
Sportiello [11]	1991	40	Retroperitoneum	10	10 days after c.s.	Excision + hormonal therapy, recurrence after 12 months, excision of recurrence + radiotherapy, rapidly second recurrence, complete response for 27+ months with hormonal treatment
Firoozmand [15]	2001	27	Ileoanal pouch	17	23 weeks of gestation	At 23 weeks gestation complete resection, outcome not reported
Wang [9]	2006	27	Neck	2	2nd month of gestation	One month after complete excision no recurrence
Sun [12]	2007	28	Mesentery	12	Immediately after c.s.	One year after complete excision no recurrence
Tankshali [16]	2011	28	Pelvis (retroperitoneal)	12	During c.s.	One year after complete excision no recurrence
Ilhan [13]	2012	22	Mesentery	7	2 months postpartum	Radical excision along with small bowel segment, outcome not reported
Present case	2013	31	Transverse mesocolon	33	3 weeks postpartum	Fifty-four months after resection no recurrence

c.s. = Caesarean section.

An essential issue is how to counsel women who have been diagnosed with a pregnancy-associated desmoid and subsequently wish to have a child. Only a small number of case reports provide some data regarding this issue (Table 2). During subsequent pregnancy an untreated pregnancy-associated desmoid tumour of the abdominal wall demonstrated volumetric increase of the tumour, necessitating surgical resection in one patient [16], while there was significant regression without treatment in another case [17]. As in the present case, the only three other patients reported with a subsequent pregnancy after surgical treatment for their pregnancy-associated desmoid demonstrated no recurrence or second desmoid tumour [18,19]. Hence, a subsequent pregnancy does not necessarily seem to be a risk factor for recurrent or new disease. Nevertheless, a follow-up period of at least 2 years before planning a subsequent pregnancy may be advisable, since the median time to desmoid recurrence after resection is approximately 1 to 2 years, as is reported in large series [4,20-22].

**Table 2:** Reports of patients diagnosed with pregnancy-associated desmoid tumour and subsequent pregnancy

**First author**	**Year**	**Patient’s age (years)**	**Site of origin**	**Size (cm)**	**Diagnosis and treatment**	**Subsequent pregnancy and outcome**
Caldwell [17]	1976	26	Abdominal wall	17.5	Shortly after birth of fourth child, observation	Almost complete regression in size during and after subsequent pregnancy
Ezra [19]	1990	35	Abdominal wall	2, 7 and 4	Recurrence after prior excision, wide resection of the area with 3 local recurrences	Subsequent pregnancy >2 years after resection. No new recurrence during and shortly after pregnancy
Way [18]	1999	28	Abdominal wall	4	Immediately after birth of second child, wide resection	Miscarriage, delivery of healthy child and abortion respectively 15, 24 and 39 months after resection. No recurrence 60 months after resection
		28	Abdominal wall	2	Twelve months after pregnancy, wide resection	Subsequent pregnancy 13 months after tumour excision. No recurrence 46 months after resection
Galeotti [16]	2006	31	Abdominal wall	10	After first pregnancy, observation	Increase in diameter from 6 to 10 cm during second pregnancy, excision of the tumour after transvaginal delivery. No data of further follow-up
Present case	2013	31	Transverse mesocolon	33	Three weeks postpartum, resection	Delivery of twins 26 months after resection. No recurrence 54 months after resection

## Conclusions

Although pregnancy-associated desmoid tumours are almost exclusively located in the abdominal wall, desmoid tumour should be included in the differential diagnosis of intra-abdominal tumours detected during or shortly after pregnancy. From the sparse literature data and from the case herein presented it seems that subsequent pregnancy is not necessarily a risk factor for recurrent or new desmoid tumour. Based on the median time to eventual desmoid recurrence after resection, it may be advisable for the patient to allow for a 2-year follow-up period before planning an eventual subsequent pregnancy.

## Consent

Written informed consent was obtained from the patient for publication of this case report and any accompanying images. A copy of the written consent is available for review by the Editor-in-Chief of this journal.

## Competing interests

The authors declare that they have no competing interests.

## Authors’ contributions

EdB and ED collected the information, reviewed the literature and wrote the manuscript. EdB, ED, EG, EGC and JM participated in analyzing the data. EG carried out the pathological studies and provided the histological figure. EGC interpreted and provided the radiological images. EdB and JM edited the article. All authors read and approved the final manuscript.
